# Enablers and hindrances for longer-term abstinence in opioid dependent individuals receiving treatment with extended-release naltrexone: A Norwegian longitudinal recovery trial (NaltRec study)

**DOI:** 10.1016/j.conctc.2021.100728

**Published:** 2021-02-10

**Authors:** B.M. Weimand, K.K. Solli, W.H. Reichelt, L. Tanum

**Affiliations:** a Mental Health Services, Akershus University Hospital, Lørenskog, Norway; b Center for Mental Health and Substance Abuse, University of South-Eastern Norway, Drammen, Norway; c Norwegian Centre for Addiction Research, University of Oslo, Oslo, Norway; d Vestfold Hospital Trust, Toensberg, Norway; e Faculty of Health Sciences, OsloMet—Oslo Metropolitan University, Oslo, Norway

**Keywords:** Extended release naltrexone hydrochloride, Opioid dependence, Recovery, Multicenter clinical trial, Cost-benefit

## Abstract

Opioid-dependence is a comprehensive, relapsing disorder with negative individual, - family, - and societal consequences. Recovery is difficult to achieve. Research has shown reduced substance use and improved health- and psychosocial factors with extended-release naltrexone (XR-NTX) treatment. Pharmacological treatment should include psychosocial interventions to improve longer-term recovery. This study explores how voluntary monthly treatment with extended-release naltrexone hydrochloride (Vivitrol®) will influence longer-term recovery, health and psychosocial relationships in opioid-dependent patients. Close relatives’ experiences and societal costs will be assessed. This Norwegian naturalistic, multicenter, open-label study includes 150 opioiddependent patients. Patients are assessed every four weeks for 24 weeks, with 28 weeks optional follow-up treatment-period, and at three, six and 12 months posttreatment. Controls are opioid-dependent patients enrolled in Opioid Maintenance Treatment programs (n = 150). Data on recovery will be collected from participants, close relatives, and community health service providers. Genetic analyses of major signaling pathways and national registries on prescriptions and health care use will be analyzed. Recruitment period is September 2018 to September 2020. The assessment of medical, psychological, relational and societal factors may provide novel in-depth knowledge on the complexity of personal recovery-processes. The results are expected to have impact on priorities in treatment and follow-up for opioid dependent patients.

## 1 Introduction

Opioid dependence is a chronic, relapsing disorder carrying numerous negative consequences for the individual, their families and to society [[1], [2], [3]]. Opioid maintenance treatment (OMT) is the most widely implemented treatment option and has shown to reduce the risk of overdose deaths, use of illicit substances and to improve the patients’ health and psychosocial situation [[4], [5], [6], [7]]. Also non-opioid treatment options are available, but continued abstinence is difficult to maintain [[8], [9], [10], [11], [12]]. Injectable extended release naltrexone (XR-NTX) is a treatment available only in the US, Russia and Ukraine. However, our research group has previously performed a randomized controlled XR-NRX trial in Norway on opioid dependent patients [13].

The results were consistent with other studies of XR-NTX [[14], [15], [16], [17], [18], [19], [20], [21], [22]] showing reduced use of opioids and other illicit substances, and an improvement on psychosocial variables [13,[23], [24], [25]]. However, treatment strategies, including treatment with XR-NTX, should include psychosocial support to improve longer-term individual recovery [9,[26], [27], [28]].

In the project group's previous RCT on XR-NTX, study participants as well as the user organizations emphasized the importance of investigating in more detail the psychological aspects of opioid receptor blockade. Supportive networks, good housing conditions and employment are factors known to facilitate abstinence and recovery [8].

Substance addiction also induces an increased risk of negative outcomes [[29], [30], [31]] regarding relational conflicts [[32], [33], [34], [35]], unstable living conditions, mental [36] and -physical [34] health problems in close relatives. A better balance between risk-and protective factors for the patient and the family members should therefore be taken into account during treatment.

How the family experience hindering and facilitating factors for the opioid dependent member's abstinence and recovery process during treatment with XR-NTX has not yet been researched. The comprehensive nature of addictive disorders means there is high demand for research on personal recovery [37] that include both the patient and the family perspective on experienced enablers and hindrances to the recovery process and the importance of family support.

Previous studies have assessed change in somatic and mental health status in OMT patients. The most common mental health problems observed were depression, anxiety-, and personality disorders [38], while the somatic health problems were more related to infections and other complications from injecting drugs [[4], [5], [6], [7]].

However, to our knowledge no previous study has assessed changes in health status and recovery in out-patients patients receiving longer term treatment with an opioid receptor blocker that induce sustained abstinence from opioids. The main aim of this study is to assess how voluntarily induced longer term abstinence from opioids influence patients’ mental and somatic health problems, and to what extent it enhances their personal recovery process.

## 2 Methods

### 2.1 Objectives

To investigate the overall aim, our primary and secondary objectives are listed below.

#### 2.1.1 Primary objectives

To investigate:

1. Whether treatment with XR-NTX, compared to OMT controls, will improve the process of recovery, with regards to adherence to treatment and counselling, adaptability to work/school, and housing situation.
2. If long time abstinence from opioids will improve mental or physical health problems in participants receiving treatment with XR-NTX compared with OMT controls.
3. The participants' perspectives on enablers and barriers to agreeing to, and continue over time with an opioid receptor blocking treatment (XR-NTX).
4. To perform a health-economic expenditure analysis across health, community, and criminal justice domains on participants receiving XR-NTX and in OMT controls.

#### 2.1.2 Secondary objectives

To investigate:

1. The effectiveness and safety of XR-NTX with regards to the use of illicit opioids and other illicit substances.
2. The retention in treatment over 24 weeks XR-NTX treatment period and an optional 28 weeks treatment period.
3. The frequency and type of adverse events (AEs) in XR-NTX participants from baseline to week 52 and 3 months after treatment.
4. “The prevalence of Attention Deficit Hyperactivity Disorder (ADHD) among XR-NTX participants will be based on an inventory in combination with genetic analyses. Furthermore, we will investigate the connection between ADHD and the use of illicit drugs, and the type and level of services needed by this group of patients to promote recovery on a wide range of life domains."
5. The conceptualization of changes in mental and somatic health status, before and after longer-term abstinence from opioids in XR-NTX participants.
6. XR-NTX participants' self-reported needs, met or unmet, during the recovery process.
7. If long term abstinence from opioids and other illicit substances, will enable the XR-NTX participant to take on tasks previously found to be too difficult mentally or physically in former recovery settings.
8. The impact of relational/familial factors on adherence to treatment, use of illicit substances and degree of recovery in XR-NTX participants.
9. Possible genetic markers for opioid dependence in cooperation with other research groups.
10. Registry data on mortality, hospitalization, use of health care services, prescribed medication and recidivism (criminal activity) from baseline to week 52 post treatment in XR-NTX participants and for a similar period in OMT controls.
11. Up to 52 weeks post-treatment efficacy of XR-NTX on the current use of illicit substances and social status including employment, housing, and familial situation.

### 2.2 Design and setting

This is a naturalistic, multicenter, open-label, trial on treatment with extended-release naltrexone hydrochloride injectable suspension (Vivitrol®) (referred to as XR-NTX in this protocol article). The study period is 24 weeks with an optional 28 weeks prolongation of treatment. A total of 150 participants will be recruited for treatment, and compared to 150 controls who receive treatment with buprenorphine (-naloxone) or methadone in an OMT program. The controls will be enrolled in the final phase of the recruitment of participants, matched group-wise on age and gender. The study will focus on how treatment with XR-NTX will influence or promote the personal recovery including physical and mental health in opioid dependent persons compared to the control group.

After the baseline assessment, the XR-NTX participants will be followed-up on a 4-weekly ( ±3 days) basis by the Principal investigator or delegated site personnel. At each visit, an injection of XR-NTX will be administered. Participant recruitment started in September 2018 and will complete in September 2020.

A partially mixed, sequential, and dominant design will be used. Partially mixed and sequential means that the quantitative and qualitative elements of the project will be undertaken separately, conducted at different times and/or stages; and dominance refers to the weight on the quantitative approach to the research questions.

The trial will be performed at five hospitals (Health Trusts) throughout the Southern part of Norway; Akershus University Hospital, Sørlandet Hospital, Vestfold Hospital, Oslo University Hospital, and Haukeland University Hospital. The catchment areas currently includes close to half of the total population in Norway.

The trial is conducted in accordance with the ethical principles originated from the Declaration of Helsinki, which are consistent with the International Conference on Harmonisation (ICH) guidelines for Good Clinical Practice (GCP) and the National regulatory requirements. Registration of participant data is carried out in accordance with the General Data Protection Regulation (GDPR) and the National Personal Data Protection regulations.

Standard operating procedures (SOPs) from the Norwegian Clinical Research Infrastructures Network (NorCRIN) will be used. The Principal Investigators (PI) and delegated site personnel are trained in ICH-GCP, protocol, trial specific procedures such as Serious adverse event (SAE) reporting, Mini-International Neuropsychiatric Interview (MINI), Addiction Severity Index, European Version (EuropASI) interview, XR-NTX preparing and injection, and entering of data into the electronic case report forms (eCRF). The monitoring of the trial is performed at all sites by the independent regional monitoring authorities at Oslo University Hospital or at Haukeland University Hospital.

The number of participants per trial site is expected to vary depending on the availability of interested subjects at the different study sites. The community health services from the catchment area of each of the hospitals (trial sites) will be invited to participate in the trial.

We will also examine how the study participants and their close family members perceive the study participants' recovery process. This exploration will focus in particular on any changes in family cohesion and social relationships, as well as their need for support and follow-up.

#### 2.2.1 User involvement

Co-researchers from the user organizations: the Drug Addicts’ Organization (Rusmisbrukernes Interesse Organisasjon, RIO) and Network for Medically Assisted Rehabilitation (proLAR, previously LAR-Nett Norge), has taken part in every step of the project. They are full members of the study steering committee, where they are being regularly updated on, and give inputs to, developments in the trial as it progresses. Co-researchers are invited to take part in data collection, analyses and discussions of the findings, and in scientific and popular publications.

### 2.3 Characteristics of XR-NTX participants

This trial has two categories of participants: individuals with opioid addiction and their relatives. The XR-NTX participants will be recruited through the participating sites, and the relatives will be recruited through the XR-NTX participants.

#### 2.3.1 Inclusion criteria

The XR-NTX participants are men or women, age 18–65 years, with a DSM-V (Diagnostic and Statistical Manual of Mental Disorders, 5th edition) diagnosis of moderate to severe opioid dependence, Single Episode (304.00) and confirmed by the Mini-International Neuropsychiatric Interview (MINI) [39].

The exclusion criteria*,* for the purpose of assuring participants’ safety and minimizing confounding variables, are:

• Current abnormal laboratory findings or observed abnormalities that need medical attention and follow-up (including: severe hepatic (Child-Turcotte-Pugh level C) or renal failure
• Current clinically significant symptoms of progressive Acquired Immunodeficiency Syndrome (AIDS)
• Current serious respiratory debilitation
• Current severe psychiatric disorder (including current or recurrent affective disorders with suicidal behavior, psychotic disorders) using the MINI 6.0 interview [39] that need medical attention and follow-up
• Ongoing alcohol dependency as defined by the criteria in DSM V
• Known intolerance and/or hypersensitivity to XR-NTX, carboxymethylcellulose, or polylactide-co-polymers (PLG), or any other components of the diluent
• Any medical or psycho social condition that would compromise the participant's ability to fulfill the protocol visit schedule or visit requirements
• Any excluded medication at screening or anticipated/required use during the study period (including: requiring treatment with opioid medications other than investigational products)
• Having had any relation to the study drug manufacturer (Alkermes)
• Being a participant in any other trial that might affect the current study
• Females are excluded from further study participation if pregnant (i.e., positive urine and/or serum pregnancy test) and/or currently breastfeeding

Females of childbearing potential should use a highly effective method of contraception and have a pregnancy test prior to each XR-NTX injection for the duration of the study.

Inclusion criteria, Control Group: Individuals, who are eligible for participation and currently enrolled in OMT, but not interested in treatment with XR-NTX, will be asked to join the control group and consent to the collection of demographic data and registry data corresponding to that of the XR-NTX group. They will be selected by the same inclusion and exclusion criteria's waived for detoxification, contraception for female of childbearing potential, use of excluded medication, and intolerance. Controls will be used for comparison of recovery-related psychosocial outcomes and change in somatic and mental health only. There will be no comparison of any pharmacological efficacy of OMT medication to XR-NTX, or any comparison of current use of illicit substances between the groups. The freedom to decline or accept participation with no consequences for hospital treatment or follow-up services will be stressed in all information provided to potential participants and controls.

#### 2.3.2 Inclusion criteria, relatives of the XR-NTX participants

Adults or adolescents above the age of 16, who are pointed out as a close relative by the XR-NTX study participants. The XR-NTX study participant must also give consent to their designated relative's participation.

### 2.4 Processes, interventions and comparisons

#### 2.4.1 Investigational product, dosage and mode of administration

The eligible individuals will be assigned to treatment with XR-NTX, 380 mg (Vivitrol®), administered intramuscularly in the gluteus where it releases blocking amounts of naltrexone for the duration of four weeks per injection.

The investigational product (Vivitrol®) will be imported to Norway in full accordance with EudraLex - Volume 4 - Good manufacturing practice (GMP) guidelines. The Quality person under Directive 2001/83/EC (QP) service and shipment of drug will be delegated to Fischer Pharmaceutical, UK, from 2019 to Fischer Clinical Services GmBH, Germany (due to Brexit). The import license is issued by Norwegian Medicines Agency (NOMA), and the trial medication is stored and distributed to the sites by the Akershus University Hospital Pharmacy.

### 2.5 Ethics approval, registration and consent to participate

The Regional Committees for Medical and Health Research Ethics, committee South East A approved the study protocol and patient information with informed consent on March 13, 2018 (# 2018/132).

The study is approved by the Norwegian Medicine Agency (NOMA), EudraCT Code 2017-004706-18, and personal data protection representative for each of the sites.

The trial is registered on Clinicaltrials.gov # NCT03647774, first registered: Aug 28, 2018, before the first participant was included on Sep 21, 2018.

#### 2.5.1 Informed consent

Documented informed consent is obtained for all participants included in the trial before any trial specific procedure is performed. The participants will be informed about the treatment and trial procedures, data privacy, insurance, voluntary participation, and that the participants could withdraw their consent to participate in the trial at any time and without stating any particular reason.

#### 2.5.2 Opioid detoxification

The participants will undergo detoxification before receiving the first injection of XR-NTX. The purpose of detoxification is to discontinue the participant's physiological dependence on opioids, and will be performed in accordance with current Norwegian national guidelines [40] and in line with international standards. In our trial, detoxification will be provided in the enrolled hospitals' detoxification units.

#### 2.5.3 Duration of treatment

Participants eligible for the trial should voluntarily seek treatment for opioid dependence, and be referred to a detoxification period in a controlled environment of minimum 7 days for the discontinuation of all illicit substances, if needed. They will also be enrolled in the Norwegian National OMT program before discharge from a controlled environment. Participants will commence to the 24 weeks of out-patient treatment with the 380 mg/4 weeks XR-NTX fixed dose. After 24 weeks of XR-NTX treatment, the participants will be given an option to enter a 28 week follow-up treatment study.

### 2.6 Data collection

At each visit (every four weeks) data will be obtained from each participant through a structured interview, on the use of heroin and other illicit substances, physical and mental health, housing and family situation, income and other social aspects. A urine sample will be obtained at every visit and screened for opioids and other illicit substances. Fertile women will in addition be screened for pregnancy (for details, see Table 1).

**Table 1:** Study flow chart for XR-NTX participants in the 52-week treatment period and 52-weeks post-treatment period.

Visit/week	Screening	Treatment period – main study				End of Treatment (EOT) – main study	Optional follow-up study – treatment period			EOT – follow-up study	After EOT	
	V1^a^	W0	W1^b^	W4, W8, W16, W20	W12	W24_1_^c^	W24_2_	W28, W32, W36, W44, W48	W40	W52	Safety FU^b^ 3 month	W 26, W52
Informed consent	X											
Inclusion/exclusion Evaluation	X											
MINI 6.0	X											
EuropASI, medical history	X											
EuropASI short form				X	X			X	X			
EuropASI 6 month						X	X			X		
Completing Detoxification		X										
TSWLS	X			X	X	X	X	X	X	X		
ISI	X			X	X	X	X	X	X	X		
McGill Pain Questionnaire	X			X	X	X	X	X	X	X		
VAS	X			X	X	X	X	X	X	X		
SCL-25	X			X	X	X	X	X	X	X		
QPR	X				X	X	X		X	X		
FACES III	X				X	X	X		X	X		
WHOQOL	X				X	X	X		X	X		
ASRS (V1)	X					X	X			X		
ISEL	X				X	X	X		X	X		
MADRS	X					X	X			X		
SURE	X					X	X			X		
Physical examination	X											
Vital signs	X											
Blood health screen	X											
Liver enzymes (ALT, AST)	X					X	X			X		
Urine analysis	X											
Pregnancy test (prior to XR-NTX injection)		X		X	X		X	X	X			
Concomitant meds.	X			X	X	X	X	X	X	X		
AE - active screening			X	X	X	X	X	X	X	X	X	X
XR-NTX injection		X		X	X		X	X	X			
End-of-treatment (EOT) for all participants						X				X		
Urine/saliva sample (drugs)		X	X	X	X	X	X	X	X	X		
Optional blood sample (genetic biomarkers)		Performed during the study period										
Follow-up interview^e^												X
Qualitative interviews^d^		Performed during the study period										

Visit 1 (V1) = screening before inclusion – leading to consideration of eligibility. V2 (week 0 (W0)) = study inclusion, and first injection of XR-NTX. V3 (W1) = telephone call one week after the first injection. V4 (W4). Second injection and follow-up screening.

a The screening period of 30 days could be extended when unforeseen incidence occurs that delay the detox period. Eligibility should then be confirmed by Coordinating Investigator.

b Visit 3 can be performed as a phone call, and the safety follow-up visit can be performed as a phone call or from the Medical Records for patients including inn the OMT.

c Visit 9_1_ is performed for the participants that do not enter the follow-up study, Visit 9_2_ is performed for the participants that enter the 28 weeks follow-up study.

d Sub-study A; 40 of the 150 study participants: 20 who stay in treatment for more than 12 weeks and 20 who receive at least one injection XR-NTX but drops out before 12 weeks. The interviews will be performed after 12 weeks of treatments or within 30 days after drop-out.

e Selected questions from the questionnaires EuropASI, TSWLS, VAS, and participation in OMT, to receive information about maintenance of opioid abstinence and recovery process.

Individual, semi-structured qualitative interviews will be performed to investigate the participants’ perspective on enablers and barriers to agreeing to and remaining in treatment with XR-NTX, including possible emotional reactions and psychosocial implications of treatment. Approximately 40 of the 150 participants will be interviewed: 20 who stay in treatment for more than 12 weeks, and 20 who receive at least one injection with XR-NTX but drop out before 12 weeks. However, we will consider extending the time frame for dropping out from 12 to 18 weeks if we do not manage to include 20 participants for interview within the described time frame.

A questionnaire to assess the XR-NTX participants’ recovery process will be sent to the community health services from each catchment area that facilitate the recovery process involving community- and specialized health services.

After end of treatment, regardless of treatment duration, (after 24 or 52 weeks, respectively), all participants will undergo a brief interview by a study worker to assess the current illicit drug use and different aspects of their social situation. These data may be crucial for determining the post-treatment efficacy of XR-NTX.

We will obtain an optional saliva sample for genetic analyses to identify possible genetic markers of deviant monoaminergic expressions and other genetic features typical for ADHD spectrum disorders. Such markers may predict the outcome of treatment with XR-NTX and identify any study outcome differences between those with and without markers of ADHD spectrum disorders.

*The control group* will be assessed at baseline, after 24 weeks and after 52 weeks only. They will be asked to respond to the items of EuropASI modified version, the MINI 6.0, an inventory assessing ADHD and provide a saliva sample for genetic analysis. They will also be assessed by the relevant community health services on their longer-term recovery process at week 24 and at week 52.

*XR-NTX participants' relatives:* We will obtain data on close relatives’ conceptions regarding the recovery process of their opioid addicted family member: possible changes in familial cohesion and social relationships, and their own need for support and follow-up. A structured questionnaire will be sent to one participant-designated close relative per participant; and three focus group interviews will be performed, each with 6–8 close relatives of the participants.

*Health-economic data:* we will obtain data on the cost of XR-NTX treatment in participants compared to OMT in the controls. Since the cost of medication is only one part of the total health cost of opioid dependence, we will try to estimate the total cost of health- and social services for this group of participants based on national registry data, hospital records and records from General physician (GPs) and social workers. Registry-based information will be collected based on participants’ personal identity number (PIN). Information on the XR-NTX participants and controls will be compared to randomly selected individuals from the National OMT registry matched for age, gender and time period of treatment. Expenditure estimates will utilize updated figures on treatment costs from trial sites, the Norwegian Ministry of Health, The Norwegian Ministry of Justice, and the Norwegian Welfare and Benefits Administration. Models from previous expenditure estimates [41] will be evaluated and improved or updated as necessary.

For bringing together the data from these different sources and for performing the health economic analysis, we will use decision-analytical modeling [42]. Only the cost of the alternatives will be compared. To handle uncertainty we will both use deterministic and probabilistic sensitivity analysis.

#### 2.6.1 Instruments

• The **MINI** is a structured screening interview for DSM-V diagnoses [39,43] is being used at baseline for evaluating the inclusion and exclusion criteria's.
• The Addiction Severity Index, European Version (**EuropASI)** version 5 structured interview is being used to assess demographics, physical health, work & education, substance use & treatment, criminal activity, and social functioning [44]. The history is assessed during the first EuropASI interview, administered at screening, while follow-up interviews (each 4 weeks for participants in the XR-NTX group) will assess present functioning only. At week 24 and at week 52 EuropASI 6 months version will be applied for participants and controls.
• The Montgomery-Aasberg Depression Rating Scale (**MADRS**) is a clinician rated symptom scale for depression [45,46].
• Substance use recovery evaluator (**SURE)** is a Patient Reported Outcome Measure for recovery from drug and alcohol dependence [47].
• The Temporal Satisfaction With Life Scale (**TSWLS**), 5-items scale, measures differences in degree of enjoyment and satisfaction [48].
• Insomnia Severity Index (**ISI)** [49], 5-items scale, is a measure of sleep difficulties.
• Craving for heroin & treatment satisfaction (**VAS**) measure a) craving for heroin b) the extent to which participants are satisfied with their current treatment and c) the extent to which they would recommend XR-NTX treatment to a friend [[50], [51], [52]].
• The Hopkins' Symptom Checklist 25 **(SCL-25)** [53] measure the severity of mental distress. The SCL-25 preserves the depression and anxiety items from the original 90-item Hopkins' Symptom Checklist.
• The Questionnaire about the Process of Recovery **(QPR**) [54], assessed recovery. The modified version for relatives, developed for Norwegian relatives [55], is designed to assess relatives' experience of the service user's recovery process.
• The Interpersonal Support Evaluation List (**ISEL**), assesses social support in both daily life and crises [[56], [57], [58], [59]].
• The Family Cohesion Subscale (**FACES-III**) [60,61] assesses relations among family members and the degree to which family members feel separated from or connected to their family. The Norwegian version has been validated and adequate psychometric properties established [59].
• The World health Organization Quality Of Life Scale (**WHOQOL**) measures multidimensional quality of life and is considered as the “gold standard” in the field. The brief version (WHOQOL- BREF) containing 24 questions measures sensory abilities, autonomy in the past, present and future activities, and social participation was used [[62], [63], [64]].
• The Adult ADHD Self Report Scale **(ASRS)** is being used for screening and diagnosis of ADHD spectrum conditions in adults [65].

See Table 1, Table 2 for the distribution of instruments during the trial and between participants and close relatives, respectively.

**Table 2:** Study flow chart for close relatives.

Data collection		
Instruments used in questionnaire	Qualitative interviews	Performed when
QPR (modified for relatives)		During study period
ISEL		During study period
FACES-III		During study period
	Focus groups	After 20 weeks of XR-NTX treatment of the patient
	Individual	After 20 weeks of XR-NTX treatment of the patient

### 2.7 Data management

The Clinical Data Management System (CDMS) including the electronic case report forms (eCRF) used in this trial is Viedoc™, a FDA Code of Federal Regulations 21 Part 11 compliant data system. The setup of the trial specific eCRF was performed by Clinical Trial Unit (CTU), Oslo University Hospital. The designated and trained trial personnel are entering the data required by the protocol into the eCRF. The Investigator is responsible for assuring that data entered into the eCRF is complete, accurate, and that entry is performed in a timely manner. The signature of the investigator will attest the accuracy of the data on each eCRF. If any assessments are omitted, the reason for such omissions will be noted on the eCRFs. Corrections with the reason for the corrections will also be recorded. AEs will be classified according to the terminology of the Medical Dictionary for Regulatory Activities (MedDRA). Concomitant medications will be classified according to the Anatomical Therapeutic Chemical (ATC) system and the Committee of Proprietary Medicinal Products (CPMP) route of administration dictionary. Illegal concomitant medication should be registered in EuropASI and not in the concomitant part of the eCRF.

Assessment of the descriptive elements in the participant's recovery process is performed using brief structured interviews with the involved health-and social workers. Interviews can be performed by telephone or teleconference, if feasible.

## 3 Statistical analyses

Power analysis will not be performed due to the exploratory nature of the trial. Both descriptive and inferential statistics will be used. Descriptive statistics, including frequency tables, graphs or scatterplots, will be provided for all primary outcomes, as well as for the changes from baseline. All statistical tests will be two-sided with a significance level of 5%, i.e., α = 0.05 unless otherwise specified. Where appropriate, model-based point estimates will be presented together with their 95% confidence interval. Missing data will be imputed using a Last Observation Carried Forward (LOCF) approach [66]. A step-wise sequential testing procedure will be used for handling multiple comparisons to preserve an overall significance level of 0.05. The research questions are in rank order. If at any step the p-value is > 0.05, any subsequent hypotheses will not be further tested.

The outcome variable will be analyzed using parametric or non-parametric (Kruskal-Wallis) analysis of variance (ANOVA), or regression model as appropriate, including treatment, study site and baseline frequency of opioid use as explanatory variables. Study sites will be treated as a random effect while all other explanatory variables will be treated as fixed effects. Changes from baseline to every assessment will be analyzed similar to the primary objective.

Incidence rates will be calculated for all AEs including SAEs leading to discontinuations or deaths. Reasons for premature discontinuation will be registered. Safety variables that evaluate physical examinations, laboratory assessments and vital signs will be analyzed by means of descriptive statistics, frequency tabulations, and graphical displays as appropriate. For all participants, physical examination and laboratory assessment will be performed as part of study enrolment.

All quantitative data analyses will be performed using at least one of the following analyses sets:

• The intention-to-treat (ITT) population will include all participants who were included to receive XR-NTX, regardless of whether first treatment dose was received or not. This population includes all who drops out regardless of duration of participation.
• The modified intention-to-treat (MITT) population (Full Analysis Set) will include all participants who received at least one dose of XR-NTX and who have at least one valid assessment. Data from the MITT population will be used for analysis of the effectiveness objectives.
• The safety population will include all participants who took at least one dose of XR-NTX classified according to the treatment actually received.
• The per-protocol (PP) population is a subset of the MITT population, and will include participants who completed the XR-NTX treatment with no major protocol violations or deviations affecting effectiveness. Data from this population will be used as a consistency check for analysis of the primary and secondary objectives.

## 4 Qualitative analyses

The qualitative data from the individual interviews will employ two different analyses:

A *phenomenographic analysis* [[67], [68], [69], [70]] to explore variations in the participants’ conceptions of the research questions, and a *manifest content analysis* with both a deductive and inductive approach, and according to the three phases described by Elo and Kyngäs [71]: the preparation phase, the organizing phase, and the reporting phase. The deductive analyses will follow key themes regarding the research questions established from systematic or scoping reviews, followed by inductive analyses on additional dimensions or themes deriving from the data. The analyses will be performed by the researchers and co-researchers.

The qualitative data from the focus group interviews with family members will be carried out with a phenomenographic analysis [[67], [68], [69], [70]] to explore variations in the relatives’ conceptions of the research questions.

Trustworthiness in the qualitative studies will be ensured by the use of Guba's (1981) [72] four actions: *Credibility* will be strengthened by the use of open approaches in the data collection, and time will be spent to allow participants to add relevant experiences and conceptions. *Transferability* will be sought by describing the data collection and the analyzing process. The same introductory and additional questions will be posed to the different participants, to ensure *dependability*. Comparisons of the study's findings with other studies as well as having discussions about possible preconceived notions of the material in the research team will be carried out to strengthen the *confirmability.*

## 5 Discussion

This article describes a trial on treatment with the extended-release naltrexone (XR-NTX), an opioid receptor blockage currently being administered to the participants of this Norwegian multi-center trial. The trial was developed in collaboration with user organizations represented both in the steering committee and as active co-researchers. This collaboration has opened up several ways to understand the importance of this type of treatment, and is continuously providing useful insights throughout the study.

Based on the current knowledge gap regarding possible hindrances and facilitators of such treatment from the perspective of persons with opioid dependency, this trial will undoubtedly provide important information. The complex composition of medical, psychological, relational and societal factors included in this trial may provide sufficient in-depth knowledge to understand the needs that opioid dependent persons may have for follow-up and support in relation to abstinence over time. Furthermore, the experience and knowledge of the participants’ immediate family can provide important information on how to optimize the recovery process. The necessity of including relatives in treatment and follow-up, both for the participant and for the family, has received increasing focus from health authorities internationally.

However, there are several challenges related to the implementation of this study. Firstly, we anticipate that making decisions regarding enrolment in the study, staying in treatment, and staying abstinent, will be demanding for the study participants. To meet this challenge, we have received ethical approval to actively seek out trial participants and even contact the health services and registries to track them. To get in contact with the participants after the medical treatment period might also pose challenges, since those who have relapsed may have moved away or otherwise have changed their contact details. As only age and gender are matched between participants and controls, it should be taken into consideration that imbalance may occur in other confounding factors.

Trials involving medical treatment like ours will always include the possibility of causing AEs, which hence will be closely monitored. We have also gained expertise in what measures to take in order to secure participants should anyone experience such events. Based on experience, AEs occurring during the first weeks of treatment will often be related to late onset abstinence symptoms. XR-NXT is regarded to have a rather benign safety profile. Our research group have already described the side effect profile of XR-NTX in a previous study, which was in line with other relevant studies, and thus well known.

Our clinical experience with XR-NTX is that participants can undergo both surgical treatment and other medical treatment procedures without stopping the XR-NTX treatment. Among other factors, this is due to the opioid-free anesthesia available in Norwegian hospitals.

Rigorous evaluation of the different effects from this trial will be reported in scientific journals and disseminated at conferences for health authorities, clinicians, researchers, and service users. Being the first study primarily focusing on recovery in opioid dependent participants receiving XR-NTX in a naturalistic setting in Western Europe, results are expected to have impact on policy makers both nationally and in other countries with similar health care systems guidelines for treatment. We also expect the results from this trial to have an impact by informing clinicians about this treatment option for opioid dependence and for whom this treatment may be suitable. As long-term maintenance of abstinence constitutes a new option for recovery, information on participants’ reaction to and coping with such a medication is highly needed.

## Funding/Support

This work was supported by unrestricted grants from the 10.13039/501100005416Research Council of Norway (grant 269864) and the 10.13039/501100006095South-Eastern Norway Regional Health Authority (2019105). Extended-release naltrexone was provided by Alkermes at no cost in accordance with an Investigator Initiated Trial agreement. Participating sites contributed with study personnel.

## List of abbreviations

**The following abbreviations and special terms are used in this article. Abbreviation or special term Explanation**
AE: Adverse event is the development of unexpected or previously unknown undesirable medical condition, the deterioration of a pre-existing medical condition, or an abnormal laboratory value/vital sign following or during exposure to a pharmaceutical product, whether or not considered causally related to the product
ADHD: Attention Deficit Hyperactivity Disorder (ICD 11)
AIDS: Acquired immunodeficiency syndrome
ALT: S-Alanine aminotransferase
ANOVA: Analysis of variance
ASRS: Adult ADHD Self Report Scale
AST: S-Aspartate aminotransferase
ATC: Anatomical Therapeutic Chemical
BP-NLX: Buprenorphine-naloxone (Suboxone©), manufactured by Reckitt-Benckiser for use in OMT
CDMS: The Clinical Data Management System, Viedoc™ is used in this trial
CPMP: Committee of Proprietary Medicinal Products
CRF: Case Report Form; a printed, optical, or electronic document designed to record all of the protocol required information to be reported to the sponsor on each trial subject
CTU: Clinical Trial Unit, Oslo University Hospital
DSM-V: Diagnostic and Statistical Manual of Mental Disorders, 5th edition
eCRF: Electronic Case Report Form, see CRF, Viedoc™ is used in this trial
EOT: End of treatment
EudraCT: European Union Drug Regulating Authorities Clinical Trials
EuropASI: Addiction Severity Index, European Version. This study uses an adapted 5th version of this instrument
FACES III: Family Cohesion Subscale
FDA: Food and Drug Administration is a federal agency of the United States Department of Health and Human Services
GCP: Good Clinical Practice
GDPR: General Data Protection Regulation
GP: General physician
GMP: Good Manufacturing Practice
ICH: International Conference on Harmonisation
ISEL: Interpersonal Support Evaluation List
ISI: Insomnia Severity Index
ITT: Intention-to-treat
LOCF: Last Observation Carried Forward
MADRS: The Montgomery-Aasberg Depression Rating Scale
MedDRA: Medical Dictionary for Regulatory Activities
MINI: Mini-International Neuropsychiatric Interview
MITT: Modified intention to treat
NOMA: Norwegian Medicines Agency, compliant with European Medicines Agency
NorCRIN: Norwegian Clinical Research Infrastructures Network, the Norwegian hub of a larger European Clinical Research Infrastructures Network - ECRIN
OD: Overdose Death; these are anticipated SAE's in the present study
OMT: Opioid Maintenance Treatment; medical treatment of opioid dependence with opioid agonists like methadone or buprenorphine. Norway has a single mandated OMT programme called LAR
PI: Principal Investigator, the investigator that is responsible for conducting the trial according to the protocol at site level. The PI can delegate some tasks to sub-investigators, study nurses or other site personnel
PIN: personal identity number
PLG: polylactide-co-polymers
PP: per-protocol population
proLAR: Network for Medically Assisted Rehabilitation (previously LAR-Nett Norge)
QPR: Questionnaire about the Process of Recovery
RCT: Randomized controlled trial
RIO: The Drug Addicts' Organization (Rusmisbrukernes Interesse Organisasjon)
SAE: Serious adverse event
SCL-25: Hopkins' Symptom Checklist, 25-item version
SOP: Standard Operating Procedure; a detailed written instructions to achieve uniformity of the performance of a specific function
TSWLS: Temporal Satisfaction With Life Scale, ‘Present’ items
V1, V2 etc: Visit 1, Visit 2, etc
VAS: Visual analog scale, scores from 0 to 10 on heroin craving, treatment satisfaction and recommendation of treatment
W0, W4 etc: Week 0, Week 4 etc
WHOQOL: World Health Organization Quality of Life Scale
WHOQOL- BREF: The brief version of WHOQOL containing 24 questions
XR-NTX: Extended-release injectable hydrochloride naltrexone, 380 mg each 4 weeks used in this study. A long-acting opioid receptor antagonist

## References

[1] Leshner A.I. (1997). Addiction is a brain disease, and it matters. Science.

[2] Emcdda (2017). European Drug Report: Trend and Developments.

[3] Unodc (2015). World Drug Report 2015.

[4] Mattick R.P., Breen C., Kimber J., Davoli M. (2014). Buprenorphine maintenance versus placebo or methadone maintenance for opioid dependence. Cochrane Database Syst. Rev..

[5] Clausen T., Waal H., Thoresen M., Gossop M. (2009). Mortality among opiate users: opioid maintenance therapy, age and causes of death. Addiction.

[6] Connery H.S. (2015). Medication-assisted treatment of opioid use disorder: review of the evidence and future directions. Harv. Rev. Psychiatr..

[7] Nielsen S., Larance B., Degenhardt L., Gowing L., Kehler C., Lintzeris N. (2016). Opioid agonist treatment for pharmaceutical opioid dependent people. Cochrane Database Syst. Rev..

[8] Hser Y.I., Evans E., Grella C., Ling W., Anglin D. (2015). Long-term course of opioid addiction. Harv. Rev. Psychiatr..

[9] Eastwood B., Strang J., Marsden J. (2017). Effectiveness of treatment for opioid use disorder: a national, five-year, prospective, observational study in England. Drug Alcohol Depend..

[10] Neale J., Nettleton S., Pickering L. (2013). Does recovery-oriented treatment prompt heroin users prematurely into detoxification and abstinence programmes? Qualitative study. Drug Alcohol Depend..

[11] Strang J., McCambridge J., Best D., Beswick T., Bearn J., Rees S. (2003). Loss of tolerance and overdose mortality after inpatient opiate detoxification: follow up study. Br. Med. J..

[12] Ravndal E., Amundsen E.J. (2010). Mortality among drug users after discharge from inpatient treatment: an 8-year prospective study. Drug Alcohol Depend..

[13] Tanum L., Solli K., Latif Z., Opheim A., Sharma-Haase K., Krajci P. (2017). Effectiveness of injectable extended-release naltrexone vs daily buprenorphine-naloxone for opioid dependence: a randomized clinical noninferiority trial. JAMA psychiatry.

[14] Comer S.D., Sullivan M.A., Yu E., Rothenberg J.L., Kleber H.D., Kampman K. (2006). Injectable, sustained-release naltrexone for the treatment of opioid dependence: a randomized, placebo-controlled trial. Arch. Gen. Psychiatr..

[15] Lee J.D., Friedmann P.D., Kinlock T.W., Nunes E.V., Boney T.Y., Hoskinson R.A. (2016). Extended-release naltrexone to prevent opioid relapse in criminal justice offenders. N. Engl. J. Med..

[16] Krupitsky E., Nunes E.V., Ling W., Gastfriend D.R., Memisoglu A., Silverman B.L. (2013). Injectable extended-release naltrexone (XR-NTX) for opioid dependence: long-term safety and effectiveness. Addiction.

[17] Krupitsky E., Zvartau E., Blokhina E., Verbitskaya E., Wahlgren V., Tsoy-Podosenin M. (2012). Randomized trial of long-acting sustained-release naltrexone implant vs oral naltrexone or placebo for preventing relapse to opioid dependence. Arch. Gen. Psychiatr..

[18] Hulse G.K., Morris N., Arnold-Reed D., Tait R.J. (2009). Improving clinical outcomes in treating heroin dependence: randomized, controlled trial of oral or implant naltrexone. Arch. Gen. Psychiatr..

[19] Kunøe N., Lobmaier P., Vederhus J.K., Hjerkinn B., Hegstad S., Gossop M. (2009). Naltrexone implants after in-patient treatment for opioid dependence: randomised controlled trial. Br. J. Psychiatry.

[20] Kunøe N., Lobmaier P., Ngo H., Hulse G. (2014). Injectable and implantable sustained release naltrexone in the treatment of opioid addiction. Br. J. Clin. Pharmacol..

[21] Lobmaier P.P., Kunøe N., Gossop M., Katevoll T., Waal H. (2010). Naltrexone implants compared to methadone: outcomes six months after prison release. Eur. Addiction Res..

[22] Lee J.D., Nunes E.V., Novo P., Bachrach K., Bailey G.L., Bhatt S. (2018). Comparative effectiveness of extended-release naltrexone versus buprenorphine-naloxone for opioid relapse prevention (X:BOT): a multicentre, open-label, randomised controlled trial. Lancet.

[23] Solli K.K., Latif Z.E., Opheim A., Krajci P., Sharma-Haase K., Benth J.S. (2018). Effectiveness, safety and feasibility of extended-release naltrexone for opioid dependence: a 9-month follow-up to a 3-month randomized trial. Addiction.

[24] Latif Z.E., Saltyte Benth J., Solli K.K., Opheim A., Kunoe N., Krajci P. (2019). Anxiety, depression, and insomnia among adults with opioid dependence treated with extended-release naltrexone vs buprenorphine-naloxone: a randomized clinical trial and follow-up study. JAMA psychiatry.

[25] Latif Z.E., Solli K.K., Opheim A., Kunoe N., Benth J.S., Krajci P. (2019). No increased pain among opioid-dependent individuals treated with extended-release naltrexone or buprenorphine-naloxone: a 3-month randomized study and 9-month open-treatment follow-up study. Am. J. Addict..

[26] Flynn P.M., Joe G.W., Broome K.M., Simpson D.D., Brown B.S. (2003). Recovery from opioid addiction in DATOS. J. Subst. Abuse Treat..

[27] Gossop M., Marsden J., Stewart D., Kidd T. (2003). The national treatment outcome research study (NTORS): 4-5 year follow-up results. Addiction.

[28] (2009). Guidelines for the Psychosocially Assisted Pharmacological Treatment of Opioid Denpendence.

[29] Velleman R. (1992). Intergenerational effects—a review of environmentally oriented studies concerning the relationship between parental alcohol problems and family disharmony in the genesis of alcohol and other problems. I: the intergenerational effects of alcohol problems. Int. J. Addict..

[30] Bancroft A., Carty A., Cunningham-Burley S., Banckett-Milburn K. (2002). Support for the Families of Drug Users: a Review of the Litterature.

[31] Hjärn A., Arat A., Vinnerljung B. (2014). Growing up with Parental Substance Abuse or Mental Illness – How Is Life as Adults? [Att Växa Upp Med Förelder Som Har Missbruksproblem Eller Psykisk Sjukdom – Hur Ser Livet Ut I Vuxen Ålder?].

[32] Zukauskas G., Ruksenas O., Burba B., Grigaliuniene V., Mitchell J.T. (2009). A study of stress affecting police officers in Lithuania. Int. J. Emerg. Ment. Health.

[33] Mitchell F., Burgess C. (2009). Working with Families Affected by Parental Substance Misuse: A Research Review: the Scottish Child Care and Protection.

[34] Dawson D.A., Grant B.F., Chou S.P., Stinson F.S. (2007). The impact of partner alcohol problems on women's physical and mental health. J. Stud. Alcohol Drugs.

[35] Orford J., Velleman R., Natera G., Templeton L., Copello A. (2013). Addiction in the family is a major but neglected contributor to the global burden of adult ill-health. Soc. Sci. Med..

[36] Ólafsdóttir J., Hrafnsdóttir S., Orjasniemi T. (2018). Depression, anxiety, and stress from substance-use disorder among family members in Iceland. Nordic Studies on Alcohol and Drugs.

[37] Slade M., Amering M., Farkas M., Hamilton B., O'Hagan M., Panther G. (2014). Uses and abuses of recovery: implementing recovery-oriented practices in mental health systems. World Psychiatr..

[38] Strain E.C. (2002). Assessment and treatment of comorbid psychiatric disorders in opioid-dependent patients. Clin. J. Pain.

[39] Sheehan D.V., Lecrubier Y., Sheehan K.H., Amorim P., Janavs J., Weiller E. (1998). The Mini-International Neuropsychiatric Interview (M.I.N.I.): the development and validation of a structured diagnostic psychiatric interview for DSM-IV and ICD-10. J. Clin. Psychiatr..

[40] (2016). National clinical guidelines for detoxification from addictive substances (In Norwegian only: nasjonal faglig retningslinje for avrusning fra rusmidler og vanedannende legemidler). Health NDo.

[41] Ravndal E., Vaglum P., Lauritzen G. (2005). Completion of long-term inpatient treatment of drug abusers: a prospective study from 13 different units. Eur. Addiction Res..

[42] Drummond M., Sculpher M., Claxton K., Stoddart G., Torrance G. (2015). Methods for the Economic Evaluation of Health Care Programmes.

[43] (2013). Diagnostic and Statistical Manual of Mental Disorders.

[44] McLellan A.T., Kushner H., Metzger D., Peters R., Smith I., Grissom G. (1992). The fifth edition of the addiction severity index. J. Subst. Abuse Treat..

[45] Bech P. (2006). Rating scales in depression: limitations and pitfalls. Dialogues Clin. Neurosci..

[46] Montgomery S.A., Asberg M. (1979). A new depression scale designed to be sensitive to change. Br. J. Psychiatry.

[47] Neale J., Vitoratou S., Finch E., Lennon P., Mitcheson L., Panebianco D. (2016). Development and validation of 'sure': a patient reported outcome measure (prom) for recovery from drug and alcohol dependence. Drug Alcohol Depend..

[48] Pavot W., Diener E., Suh E. (1998). The temporal satisfaction with life scale. J. Pers. Assess..

[49] Bastien C.H., Vallières A., Morin C.M. (2001). Validation of the Insomnia Severity Index as an outcome measure for insomnia research. Sleep Med..

[50] Krupitsky E., Nunes E.V., Ling W., Illeperuma A., Gastfriend D.R., Silverman B.L. (2011). Injectable extended-release naltrexone for opioid dependence: a double-blind, placebo-controlled, multicentre randomised trial. Lancet.

[51] Sullivan M.A., Vosburg S.K., Comer S.D. (2006). Depot naltrexone: antagonism of the reinforcing, subjective, and physiological effects of heroin. Psychopharmacology (Berl).

[52] Comer S.D., Sullivan M.A., Yu E., Rothenberg J.L., Kleber H.D., Kampman K.M. (2006). Injectable, sustained-release naltrexone for the treatment of opioid dependence: a randomized, placebo-controlled trial. Arch. Gen. Psychiatr..

[53] Derogatis L.R., Lipman R.S., Rickels K., Uhlenhuth E.H., Covi L. (1974). The Hopkins Symptom Checklist (HSCL): a self-report symptom inventory. Behav. Sci..

[54] Neil S.T., Kilbride M., Pitt L., Nothard S., Welford M., Sellwood W. (2009). The questionnaire about the process of recovery (QPR): a measurement tool developed in collaboration with service users. Psychosis.

[55] Weimand B., Israel P., Ruud T. (2014). Relatives and ACT-Team. A Study on Relatives' Experiences with ACT-Team, and ACT-Team Leaders' Experiences with Collaboration with Relatives [Pårørende Og ACT-Team. En Undersøkelse Om Pårørendes Erfaringer Med ACT-Team, Og ACT-Teamlederes Erfaringer Med Samarbeid Med Pårørende].

[56] Cohen S., Hoberman H.M. (1983). Positive events and social supports as buffers of life change Stress 1. J. Appl. Soc. Psychol..

[57] Delistamati E., Samakouri M.A., Davis E.A., Vorvolakos T., Xenitidis K., Livaditis M. (2006). Interpersonal Support Evaluation List (ISEL)--college version: validation and application in a Greek sample. Int. J. Soc. Psychiatr..

[58] Rogers E.S., Anthony W., Lyass A. (2004). The nature and dimensions of social support among individuals with severe mental illnesses. Community Ment. Health J..

[59] Hagen K.A., Ogden T., Bjornebekk G. (2011). Treatment outcomes and mediators of parent management training: a one-year follow-up of children with conduct problems. J. Clin. Child Adolesc. Psychol. : the official journal for the Society of Clinical Child and Adolescent Psychology, American Psychological Association, Division 53.

[60] Olson D.H. (1986). Circumplex Model VII: validation studies and FACES III. Fam. Process.

[61] Olson D.H., Russell C.S., Sprenkle D.H. (1983). Circumplex model of marital and family systems: VI. Theoretical update. Fam. Process.

[62] (1993). Study protocol for the world health organization project to develop a quality of life assessment instrument (WHOQOL). Qual. Life Res..

[63] (1998). Development of the world health organization WHOQOL-BREF quality of life assessment. The WHOQOL group. Psychol. Med..

[64] Feelemyer J.P., Jarlais D.C.D., Arasteh K., Phillips B.W., Hagan H. (2014). Changes in quality of life (WHOQOL-BREF) and addiction severity index (ASI) among participants in opioid substitution treatment (OST) in low and middle income countries: an international systematic review. Drug Alcohol Depend..

[65] Schweitzer J.B., Cummins T.K., Kant C.A. (2001). Attention-deficit/hyperactivity disorder. Med. Clin..

[66] Saha C., Jones M.P. (2016). Type I and Type II error rates in the last observation carried forward method under informative dropout. J. Appl. Stat..

[67] Marton F. (1981). Phenomenography — describing conceptions of the world around us. Instr. Sci..

[68] Marton F., Phenomenography T.H., Postlethwaite T.N. (1994). The International Encyclopedia of Education.

[69] Fridlund B., Hildingh C. (2000). Qualitative Research Methods in the Service of Health.

[70] Dahlgren L.O., Fallsberg M. (1991). Phenomenography as a qualitative approach in social pharmacy research. J. Soc. Adm. Pharm.: JSAP (J. Small Anim. Pract.).

[71] Elo S., Kyngas H. (2008). The qualitative content analysis process. J. Adv. Nurs..

[72] Guba E.G. (1981). Criteria for assessing the trustworthiness of naturalistic inquiries. Educ. Technol. Res. Dev..

